# Key lessons and strategies for implementing single IRB review in the Trial Innovation Network

**DOI:** 10.1017/cts.2022.391

**Published:** 2022-04-19

**Authors:** Ann R. Johnson, Megan Kasimatis Singleton, Julie Ozier, Emily Serdoz, Jennifer G. Beadles, Janelle Maddox-Regis, Sarah Mumford, Jeri Burr, J. Michael Dean, Daniel E. Ford, Gordon R. Bernard

**Affiliations:** 1 University of Utah Health Sciences Center, Salt Lake City, UT, USA; 2 Johns Hopkins University School of Medicine, Baltimore, MD, USA; 3 Vanderbilt University Medical Center, Nashville, TN, USA; 4 University of Utah School of Medicine, Salt Lake City, UT, USA

**Keywords:** IRB, Single IRB, Human Research Protection, Reliance agreement, Local context

## Abstract

The Trial Innovation Network has established an infrastructure for single IRB review in response to federal policies. The Network’s single IRB (sIRBs) have successfully supported over 70 multisite studies via more than 800 reliance arrangements. This has generated several lessons learned that can benefit the national clinical research enterprise, as we work to improve the conduct of clinical trials. These lessons include distinguishing the roles of the single IRB from institutional Human Research Protections programs, establishing a consistent sIRB review model, standardizing collection of local context and supplemental, study-specific information, and educating and empowering lead study teams to support their sites.

## Introduction

Challenges to implementation of a single IRB (sIRB) model in multisite research have been posited in the recent literature, since the mandate for use of a sIRB has been issued by both the National Institutes of Health (NIH) and the US Department of Health and Human Services (DHHS) effective in 2018 and 2020, respectively[1,2]. Such challenges include separating the role of the IRB from the responsibilities of a research institution, lack of investigator and institutional experience with the sIRB model, building relationships between researchers and the sIRB, and adequately capturing and considering contextual information from the individual research sites in the sIRB review [3−6]. The Trial Innovation Network (TIN), funded by the National Center for Advancing Translational Sciences, was established to provide operational innovation to clinical research, including sIRB review. The TIN has three sIRBs – Johns Hopkins University School of Medicine, the University of Utah, and Vanderbilt University Medical Center – that support network studies in operationalizing sIRB review. TIN sIRBs have been at the forefront of implementing sIRB methodology, supporting over 70 multisite studies that include more than 800 reliance arrangements. While TIN sIRBs are unique in their charge and resources, lessons learned to date are not unique to the TIN sIRBs. Five years after the TIN’s inception, we can share successful strategies to address the challenges proposed in the literature and describe new challenges for consideration.

## Emphasize and Distinguish the Roles of the sIRB and Institutional Human Research Protections

**Fig. 1.**
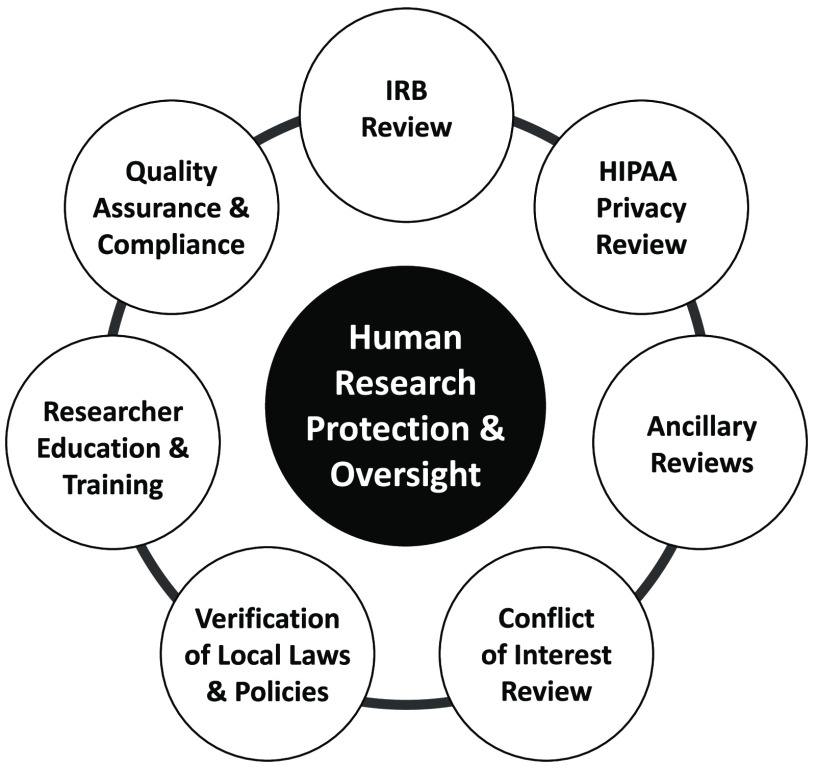
Human Research Elements of Protection & Oversight.

While an sIRB may take responsibility for the review and approval of research using criteria that are well defined in the federal regulations, the research institution must retain responsibility for the other elements of institutional Human Research Protection (HRP) and oversight (Fig. 1). This includes elements that vary widely across organizations, such as conflict of interest review, ancillary safety and resource reviews, researcher education and training requirements, quality assurance and research compliance. Ancillary safety and resource reviews may include use of local data such as EMR data, investigational drug services, radiation safety and determination of payment for services provided in clinical research protocols, and scientific reviews. It has been common for institutions to combine these elements into the responsibilities of the IRB Office, which has created difficulty when implementing the sIRB model, as the institution must find ways to separate these roles and functions. Investigators have often been unaware of this delineation of roles, resulting in confusion when trying to meet sIRB and institutional requirements. In response to this challenge, the TIN sIRBs created and used educational materials that focus on the separate roles of the sIRB and relying institution and how investigators are expected to interact with both.

The use of a reliance agreement that clearly distinguishes the roles of the sIRB from the relying institution is also critical for addressing this challenge. The TIN SIRBs utilize the SMART IRB Agreement [7] as the basis for reliance whenever possible, which provides a robust structure for understanding the roles of both the sIRB and relying institution. Some review responsibilities are flexible and can be performed by either the sIRB or the relying institution, such as HIPAA privacy review. TIN sIRBs use the IRB Reliance Exchange [8] (IREx) at Vanderbilt University Medical Center to document these case-by-case decisions in the platform’s Study-Specific Reliance Plans [9] and ensure the appropriate party understands their obligation to perform each responsibility. The TIN sIRBs also ensure that relying institutions and investigators have clear expectations for how and when to interact with the sIRBs to perform and report on their responsibilities; this is discussed further in the next section.

## Establish a Consistent sIRB Review Model

Due to national inexperience using an sIRB for multisite research, there was a great need to create a consistent, step-wise sIRB review model. The TIN sIRBs recognized that having a consistent model would help to set expectations for relying institutions and generate routine familiarity when working with the TIN sIRBs, in addition to setting a standard other sIRBs could follow. By reducing confusion and increasing familiarity, the goal was to improve the study initiation process and timelines.

The TIN sIRB review model includes the following steps (Table 1): reliance consultation, establishing reliance relationships, collection of initial local context information, sIRB review, site HRP review, sIRB approval, and site HRP activation. Participating site investigators and HRP personnel were offered education on the process when reliance relationships were being established, and education materials were posted publicly online. As this standard model was implemented and site HRP programs (HRPPs) have become familiar with the process through education and experience, the median time between when a site is invited to rely on the sIRB and when the site submits an application to the sIRB has declined from 147 days in 2018 to 95 days in 2021 (Table 2). The median time for TIN sIRB review of the main protocol and lead site has ranged from 56 to 77.5 days over the last 5 years (Table 3). Once a site application is submitted to the TIN sIRB, the sIRBs have consistently maintained an average of 12–14 days to approval of the site.

**Table 1.** Trial Innovation Network (TIN) single IRB (sIRB) review model

sIRB review model step	Description
Reliance consultation	Study personnel consult with the sIRB to determine if use of sIRB review is appropriate or required. The consultation process allows the sIRB to gather information about the proposed project in order to formulate a review plan, including timelines, and educate the study team about the process and coordination responsibilities. The discussion may also identify potential concerns impacting the sIRB review plan, such as pending FDA determinations, funding timelines, and local differences in execution of the protocol.
Establishing reliance relationships	A reliance agreement must be established between the participating site and the sIRB; the TIN generally uses the SMART IRB Agreement and may also require a letter of indemnification as an addendum. Once a master reliance agreement is in place, the relying institution can make individual reliance decisions on a study-by-study basis, as the agreement itself does not obligate the institution to rely in any case. Where the master reliance agreement allows for flexibility in the division of and execution of responsibilities on a study-specific basis, a Study Specific Reliance Plan is created by the sIRB and confirmed in the IRB Reliance Exchange IREx by a human research protection (HRP) representative of the relying institution.
sIRB protocol review and approval	The information and materials needed for a multisite sIRB submission are generally no different than a single site IRB submission. The sIRB considers the submission, evaluating the protocol according to the federal requirements as it would for any other study. *It is generally recommended that the main protocol receive review and approval before the sites pursue review and approval, as this streamlines the site review and approval process.*
Collection of local context information and site Human Research Protections (HRP) review	Institution-specific and study-specific local context information is collected in IREx from Relying Institutions for use by the sIRB during its review. Institution-specific information is collected in the IREx Institutional Profile. The study-specific information is collected via surveys and supplemental forms completed by the Relying HRP representative and site investigator. Site HRP review at the relying institution generally includes, but is not limited to, review of financial conflicts of interest; confirmation of training and qualifications for research staff; institutional reviews for safety, science, and resources; and application of local laws and policies.
sIRB site review and approval	Once the sIRB confirms the necessary elements from the local context information and Site HRP review, approval can be issued for the site if all Criteria for IRB Approval of Research [2] have been met.
Site HRP activation	Once the protocol and site are approved by the sIRB, the site HRP review is finalized and the site is activated to begin research activities.

**Table 2.** Time in days from site reliance invitation to site submission to the Trial Innovation Network single IRB

Year of site submission	Number of site submissions	Median	Minimum	Maximum	Standard deviation
2017	40	**61**	1	150	37.06
2018	172	**147**	1	649	172.82
2019	156	**129**	5	674	142.49
2020	92	**124**	2	579	111.83
2021	82	**95**	6	349	76.67

**Table 3.** Time in days from Trial Innovation Network single IRB submission to approval for the main protocol and lead site

Year of initial protocol submission	Number of submissions	Median	Minimum	Maximum	Standard deviation
2017	10	56	8	140	40.18
2018	14	77.50	27	254	65.54
2019	14	53.00	11	131	32.06
2020	11	58.00	12	189	49.53
2021	11	61.00	13	241	62.47

Despite success in establishing a clear process, some challenges remain. Some sites are still not prepared to function in relationships with sIRBs. Sites new to research – such as private practices, nonprofit and community organizations, and businesses – must secure a federal-wide assurance, delaying the reliance relationship. These sites new to research also have difficulty performing all aspects of the site HRP review, which requires additional support from more experienced sites or the sIRB. There are also sites that are uncomfortable documenting their willingness to rely on the sIRB before the sIRB has performed initial review of their site; however, without the commitment to rely and provide initial local context information, it is difficult or impossible for the sIRB to commit to conduct a meaningful review of the site. Further education is needed to facilitate relationships with inexperienced research sites; distinguish the implications of a reliance decision, sIRB approval for a site, and site HRP review completion, and resolve the different expectations of the sIRB and institution for the initial sIRB review steps and timing.

To identify early which sites may be inexperienced and require additional support to navigate the sIRB review model, it is recommended that each site’s sIRB experience be assessed as part of site selection and feasibility questionnaires, which will enable lead teams and coordinating centers to identify and budget for additional resources that may be required to support both experienced and inexperienced sites. It is further recommended that site HRPPs publicize their HRP review components and processes, such that investigators can easily access this information and prepare for the process.

## Standardize Collection of Local Context and Supplemental, Study-Specific Information

Through IREx, TIN sIRBs standardized and centralized collection of local context information via the institutional profile, an HRP survey, and an investigator survey [9]. The institutional profile, completed once and maintained by an HRP representative, documents overarching organizational information, local laws and policies, required site-specific consent language, and population characteristics. The HRP survey is used to capture study-specific information regarding the site’s conflicts of interest, standards of care, training and qualifications of local study personnel, and the results of ancillary reviews, such as radiation safety and biosafety. The investigator survey captures site differences in how the protocol may be implemented, such as consenting or recruitment differences.

Local context considerations ended up being more complex than originally anticipated, which required revisions to the local context collection tools in IREx to reach their present state described above. It also required use of supplemental, study-specific local context forms to capture information requested by the sIRB about the relying sites as part of their review. Traditional views of local context have centered on an IRB’s understanding of the social, cultural, and political attitudes and norms of the community where an institution will draw participants [5]. While this view of local context is complex in its own right and presents challenges to an sIRB for thoroughly applying the context to its review, this view is not the only local context information that needs to be gathered. Differences in institutional policies and standard practices also need to be collected and may have more of an impact on the sIRB review decisions than traditional local context information. For example, the TIN sIRBs supported several studies that were comparing a new, experimental intervention to a commonly accepted and existing treatment or procedure; however, the usual care often differed between the sites. The sIRB had to consider the risk–benefit ratio separately for different sites and consent language describing the procedures and risks differed as well. Such variations in usual care were generally not described in or accounted for in the protocol and the study design, such that the sIRB frequently discovered the information haphazardly via interactions with site personnel. As a result, the TIN sIRBs began supplementing the standard local context information with protocol- or study-specific questions to further distinguish local factors that might contribute to study review and execution at a site, as warranted by the study. To help address this challenge, investigators should think broadly about how variances in local standards of care, law, or organizational policies may impact protocol implementation and sIRB review. These variances should be accounted for in the protocol, so they may be addressed by the sIRB up front as part of its initial review rather than as each site introduces a new variance, which results in delays in site onboarding.

While the TIN sIRBs have established consistent processes to collect local information at study onset, the quality of the information received varies, delaying site onboarding. Moreover, the ability to collect relevant local information throughout the study rather than at a single time point is limited. Lastly, there continues to be a struggle with institutional preference and policy differing from the way the protocol, consent, and other materials are drafted, such as preferences over how statements in the consent document are written. This leads to delays as desired changes are negotiated. To identify early which sites may be inexperienced and require additional support to navigate the sIRB review model, the TIN sIRBs recommend that sIRB experience be standardly assessed as part of site selection and feasibility questionnaires which will enable lead teams and coordinating centers to identify and budget for additional resources that may be required to support such sites.

## Educate and Empower Lead Study Teams to Support Their Sites

Study teams, accustomed to interactions and support from their local IRBs, are now required to interact with external IRBs where the same level of communication and tailored support may not be forthcoming or even feasible. In response to this shortcoming, TIN sIRBs have developed a robust model for investigator engagement. This begins in consultation with the project’s lead study team. Expectations for sIRB review processes are established. Tailored training is offered for site study personnel via webinar and phone, or in-person where feasible. TIN sIRB coordinating center teams help sites navigate the onboarding process, providing individual support for the review of local information, and preparing materials for sIRB review. Some TIN sIRBs also enhanced their electronic submission systems to enable direct communication with site personnel. However, practical communication challenges still exist between site investigators and the TIN sIRBs, such as the time needed to build meaningful working relationships, as well as the sIRB staff needed to engage in conversations with many site study personnel.

Outside of the TIN, many sIRB offices do not have the staff capacity to support the needs of researchers at other sites. Lead study teams unaffiliated with the TIN may be unfamiliar with the demands of sIRB site coordination or they are similarly understaffed to assume the responsibilities. While TIN sIRB resources and IREx exist to help standardize and support sIRB coordination, additional consideration of how researchers coordinate the sIRB process is needed.

## New Challenges to Consider

The TIN sIRB experience has uncovered additional fundamental challenges related to sIRB review. These include lack of preparedness of research teams and organizations to operate under an sIRB model, inadequate attention to the true cost of sIRB review, and misplaced emphasis solely on measurements of efficiency to evaluate the sIRB model.

Prior to the rollout of the NIH policy, little focus was given to the critical importance of a robust educational program surrounding sIRB. Despite improved relationships, robust tools, and standardized communications, researchers and organizations remain confused about the process and purpose of sIRB review. As a result, TIN sIRBs have dedicated many hours developing educational materials and discussing the fundamentals of reliance and the sIRB review process with researchers and HRPPs. One advantage that the TIN sIRBs found was the ability to work with the same lead teams or coordinating centers over time. As there is generally a steep up-front learning curve for those responsible for submitting to the sIRB and coordinating local site completion of HRP review, the TIN sIRBs saw a measurable improvement in navigation of sIRB processes for lead teams/coordinating centers that had been through the process before. This experience highlights the importance of these roles in sIRB implementation and suggests that efficiencies may be gained when lead teams/coordinating centers become adept in managing these responsibilities.

Additionally, there has been limited public discourse on the true costs of the sIRB model. Where local IRB review systems were relatively well established, sIRB review has brought new infrastructure costs associated with system enhancement and expansion, tool development, and growth in IRB staff and membership. Research teams must hire new staff or coordinating centers to manage sIRB communications and must account for this in the direct costs of their budgets. These expenses have not been well documented, nor have they been evaluated against the cost of a traditional local review model. Additionally, budgets must include sIRB review fees, and investigators need to seek quotes for these fees up-front to include in funding applications. Though sIRB fees vary depending on the organization performing the sIRB review, fee structures commonly charge for initial and ongoing protocol and document reviews, as well as additional fees on a per-site basis. Until true costs are determined, sIRBs, relying sites, and researchers will likely remain under-resourced for sIRB review.

Finally, a dominant misconception remains that centralized IRB review is and should be faster than the use of local IRB review at each site. Not only does this emphasis on speed pose potential ethical challenges for human subject protections, it dismisses the true steps required to effectuate sIRB review. While sIRB review avoids duplicative “IRB reviews,” it does not alleviate the need for site HRP involvement with each study. Thus, the question becomes: does separating the responsibility for the IRB review and site HRP review streamline the process? While this may take years to evaluate, we must ensure our metrics capture the full time to site activation – including the site HRP review and finalization steps that lead to activation. Importantly, we must also ensure that our measurements of the success of the sIRB mandate are not simply based on time, but on the quality of human subject protections. Arguably, our greatest remaining challenge is how to keep that mission at the forefront of research considerations.

In view of the challenges and costs uncovered by the experience in the TIN, it may be reasonable to reconsider the current federal policy that requires sIRB review for studies with two or more sites [2], allowing for greater flexibility in the use of sIRB review. This could include defined options for exceptions to the sIRB requirement with appropriate justification of barriers and costs, or redefining an eligible multisite study to involve a larger number of sites (e.g., five or more sites instead of two or more sites).

## Conclusion

The TIN sIRBs have successfully overcome many challenges while implementing a sIRB review model. These lessons learned provide a solid foundation that the research enterprise can build upon to create greater innovation in this arena. As further work is performed, it will be important for all involved in sIRB implementation to report on their successes and challenges not only to strengthen the base of knowledge but also to fuel additional improvements to clinical research.
